# A91 INTERACTIONS BETWEEN PREGNANCY, DELIVERY, AND ILEAL POUCH-ANAL ANASTOMOSIS FOR INFLAMMATORY BOWEL DISEASE: A RETROSPECTIVE CHART REVIEW

**DOI:** 10.1093/jcag/gwac036.091

**Published:** 2023-03-07

**Authors:** S C House, P Tandon, K O'Connor, C Maxwell, E Kennedy, J Snelgrove, A DeBuck, M Brar, V Huang

**Affiliations:** 1 University of Toronto; 2 Mount Sinai Hospital, Toronto, Canada

## Abstract

**Background:**

Ulcerative colitis (UC) is a type of inflammatory bowel disease (IBD) that affects people in their reproductive years of life. Surgical treatment for medically refractory UC involves surgery over 2-3 stages, which includes a subtotal colectomy followed by creation of an ileal pouch-anal anastomosis (IPAA), known as a “J pouch”. The IPAA allows preservation of fecal continence and avoids the psychosocial impacts of a stoma. The IPAA procedure is a deep pelvic surgery, which may impact pregnancy outcomes. Caesarean section (C-section) delivery is often performed to avoid anal sphincter and J pouch damage from vaginal delivery. However, literature demonstrates conflicting results regarding the risks of C-section compared to vaginal delivery, including the impact on pouch function. Surveys of clinicians also report varying delivery recommendations.

**Purpose:**

To describe the delivery methods, pregnancy outcomes and postpartum course of IBD patients with IPAA at Mount Sinai Hospital.

**Method:**

A retrospective chart review is being performed for female patients at Mount Sinai Hospital (Toronto, Ontario) with a diagnosis of IBD and an IPAA. Eligible patients completed a pregnancy from January 1, 2002-February 1, 2021 post-IPAA surgery and had variables of interest accessible in their electronic medical record. Variables of interest include demographics, pregnancy history, IBD characteristics, IPAA surgery details, pregnancy outcomes, mode of delivery and characteristics, and postpartum complications. Clinical data will be presented as means, medians and frequencies. Differences between variables of interest will be evaluated with Student’s *t*-test or chi-squared test.

**Result(s):**

Three avenues of patient identification yielded 1113 patients to be screened. Inclusion criteria were met for 71 patients and chart review is complete for 36 patients who had a total of 53 pregnancies and delivered 56 babies. Most patients (49%) had a two-stage IPAA surgery, 21% required a three-stage surgery and 30% were undocumented. Most patients’ (55%) IPAA was created through laparotomy, while 13% was through laparoscopic procedure and 32% was undocumented. Seventy-four percent of deliveries were through C-section (75% of primiparous), 69% of which were indicated to protect the patient’s J pouch and 31% for an obstetrical indication. The remaining 26% of deliveries were vaginal, 29% of which were assisted with forceps or vacuum, 57% had tears (37.5% first-, 37.5% second-, and 25% third-degree) and 50% had an episiotomy.

**Conclusion(s):**

At Mount Sinai Hospital, most IBD patients with an IPAA who completed a pregnancy had a history of laparotomy to create their IPAA. Most patients (74%) with IBD and an IPAA are delivering through C-section, and mainly to protect their J pouch, which is in line with reports in the literature. Most patients had a tear or episiotomy during vaginal delivery. Rates of third-degree tears may be higher than in the general population. Trends will be further elucidated with advancement of the study.

**Disclosure of Interest:**

None Declared

